# Supplementary material for: Microbiomes of Blood-Feeding Triatomines in the Context of Their Predatory Relatives and the Environment
Source: Microbiol Spectr. 2023 Jun 8;11(4):e01681-23. doi: 10.1128/spectrum.01681-23 (PMC10433993; doi:10.1128/spectrum.01681-23)
Supplement: Supplemental file 1 — Figures S1-S7. Download spectrum.01681-23-s0001.pdf, PDF file, 1.9 MB [file spectrum.01681-23-s0001.pdf]

Supplementary Figures

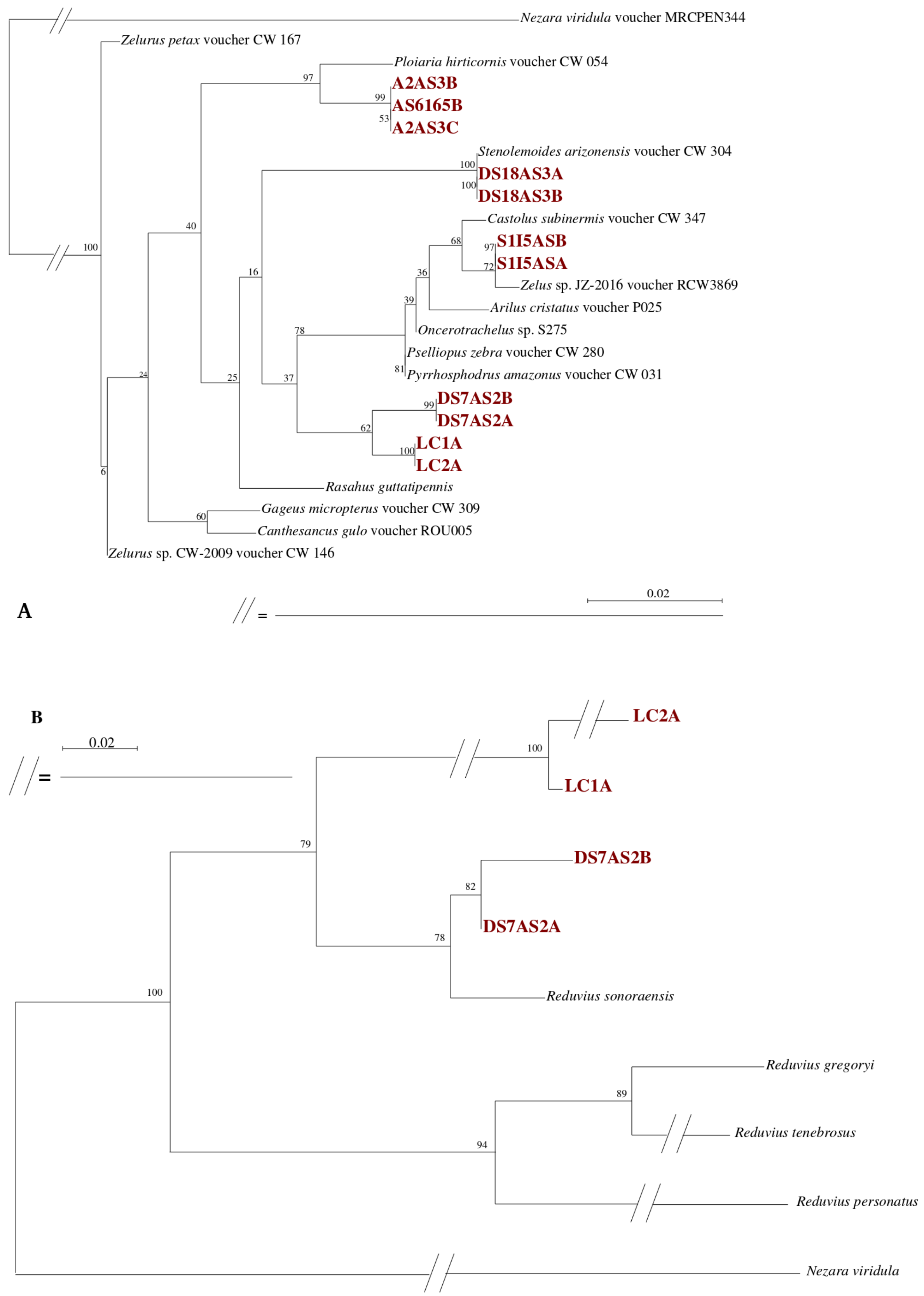

Supplementary Figure S1: Taxonomy assignment of sampled assassin bugs. Phylogeny inference on 18S rRNA gene sequences (A) did not allow for complete species determination. 16S rRNA gene-based inference was performed for the remaining four samples (B).

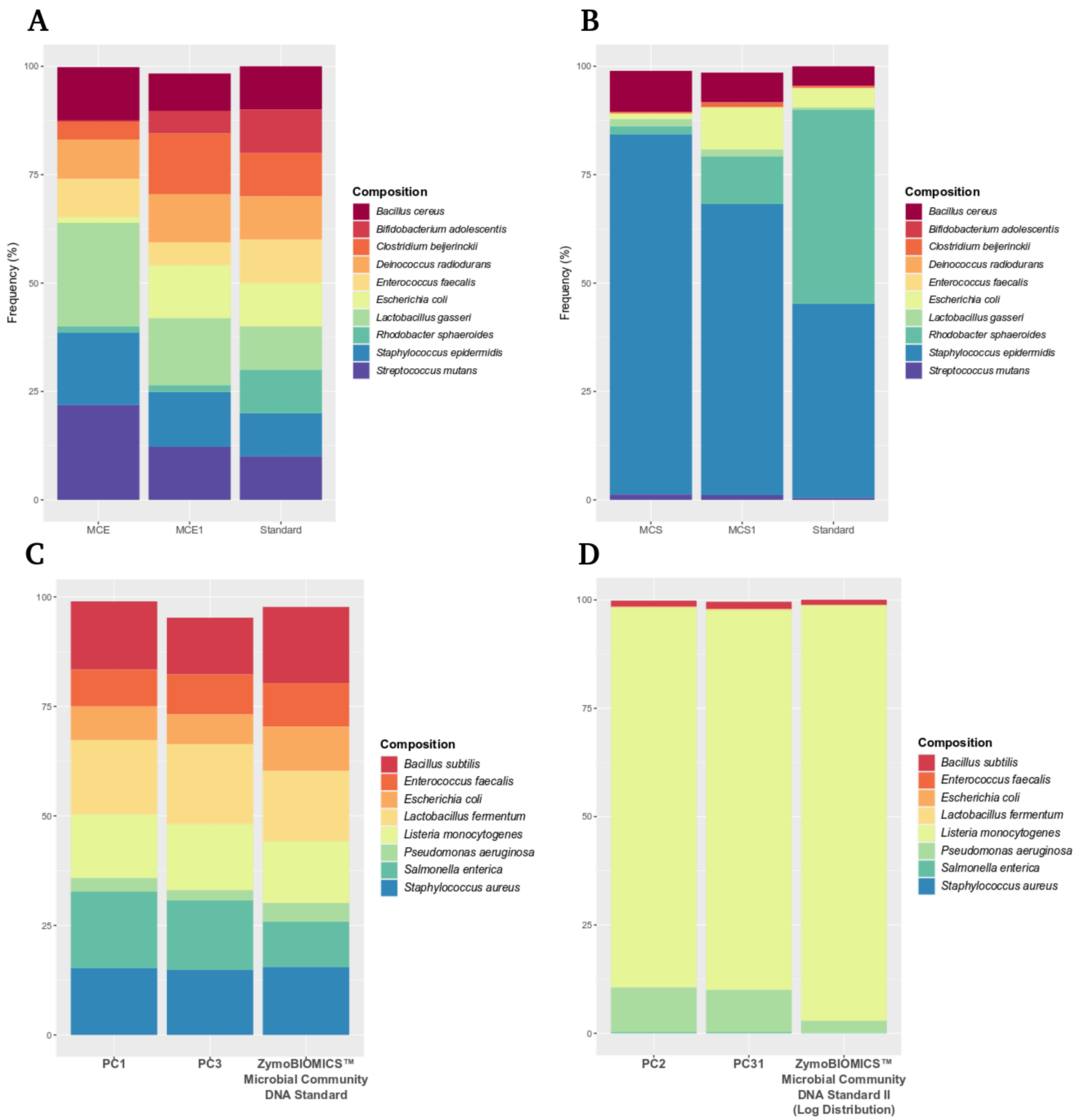

Supplementary Figure S2: Frequencies of taxa in the positive controls as recovered by 16S rRNA amplicon sequencing. In every plot, columns on the right represent the frequencies declared in the commercially purchased DNA templates. **A:** ATCC® MSA-1000™ Standard, **B:** ATCC® MSA-1001™, **C:** ZymoBIOMICS™ Microbial Community DNA Standard II, **D:** ZymoBIOMICS™ Microbial Community DNA Standard II Log Distribution. Overall, frequencies are coherent, albeit under-sequencing of *Rhodobacter sphaeroides* and over-sequencing of *Staphylococcus epidermidis* and *Streptococcus mutans* (A, B), and over-sequencing of *Pseudomonas aeruginosa* (D).

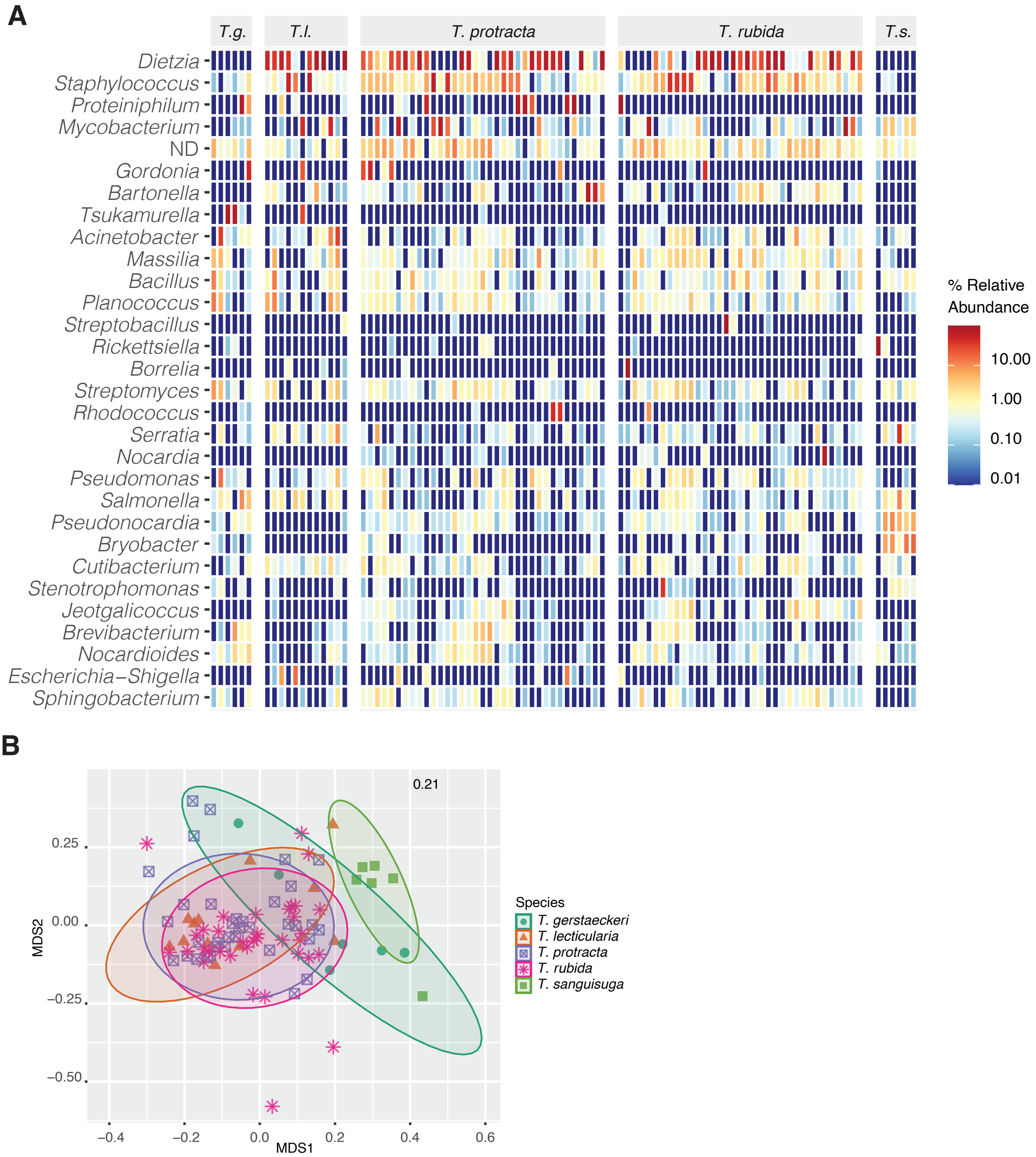

Supplementary Figure S3: Microbial profiles of five *Triatoma* species. **A:** Heat map for first 30 most abundant genera across the *Triatoma* samples. **B:** NMDS ordination for based on Bray-Curtis dissimilarities. The number in the right upper corner indicates the stress value.

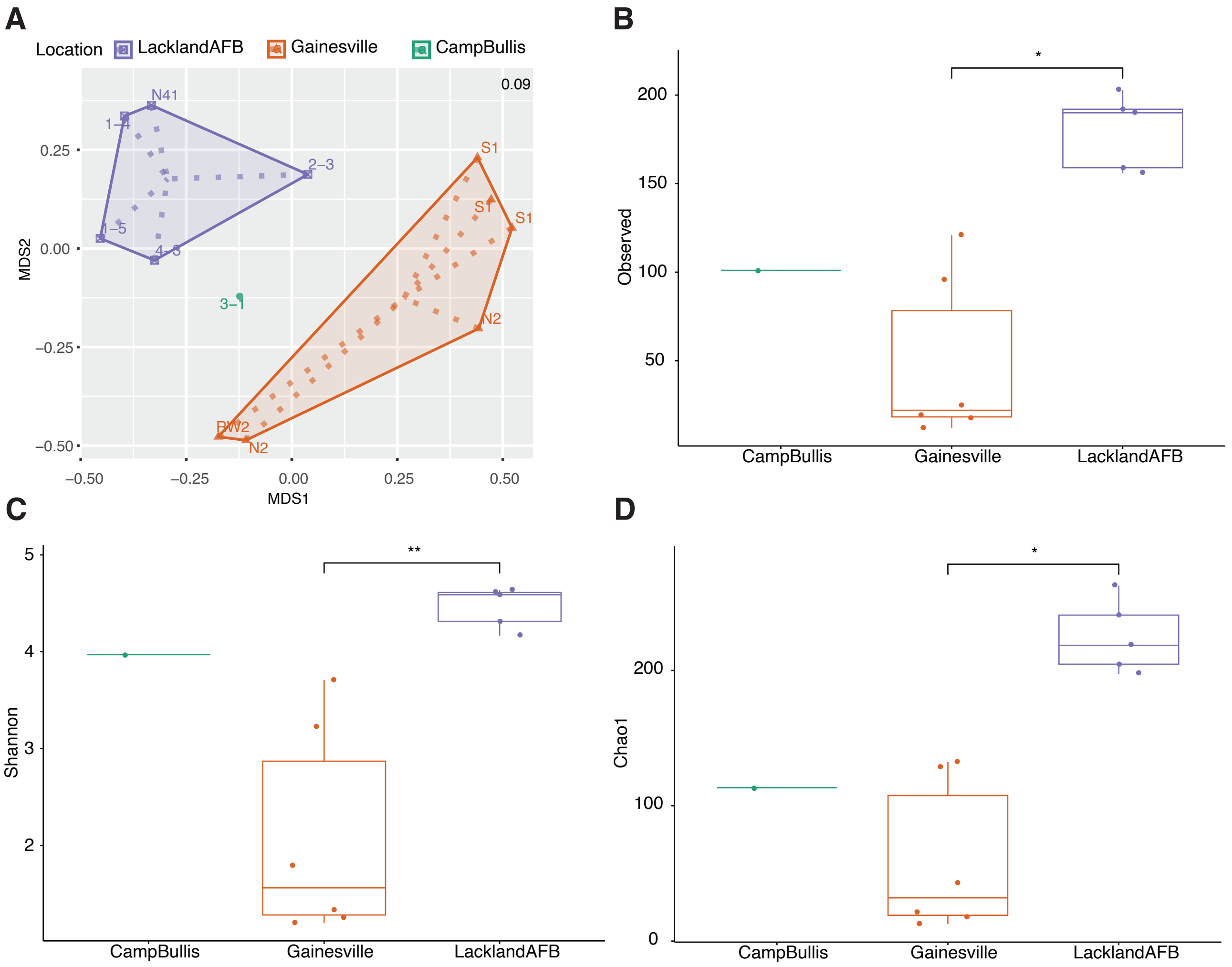

Supplementary Figure S4: Microbial profiles of nest materials. **A:** NMDS ordination for nest material samples based on Bray-Curtis dissimilarities. The number in the right upper corner indicates the stress value. Designation of each nest reflects the codes used in the heatmaps in Figure 1. **B:, C:, D:** Alpha diversity measures for nest material samples from different location. Asterisks stand for significant differences as identified by Dunn's Kruskal-Wallis multiple comparisons (Supplementary Table S1-DATASHEET S10)

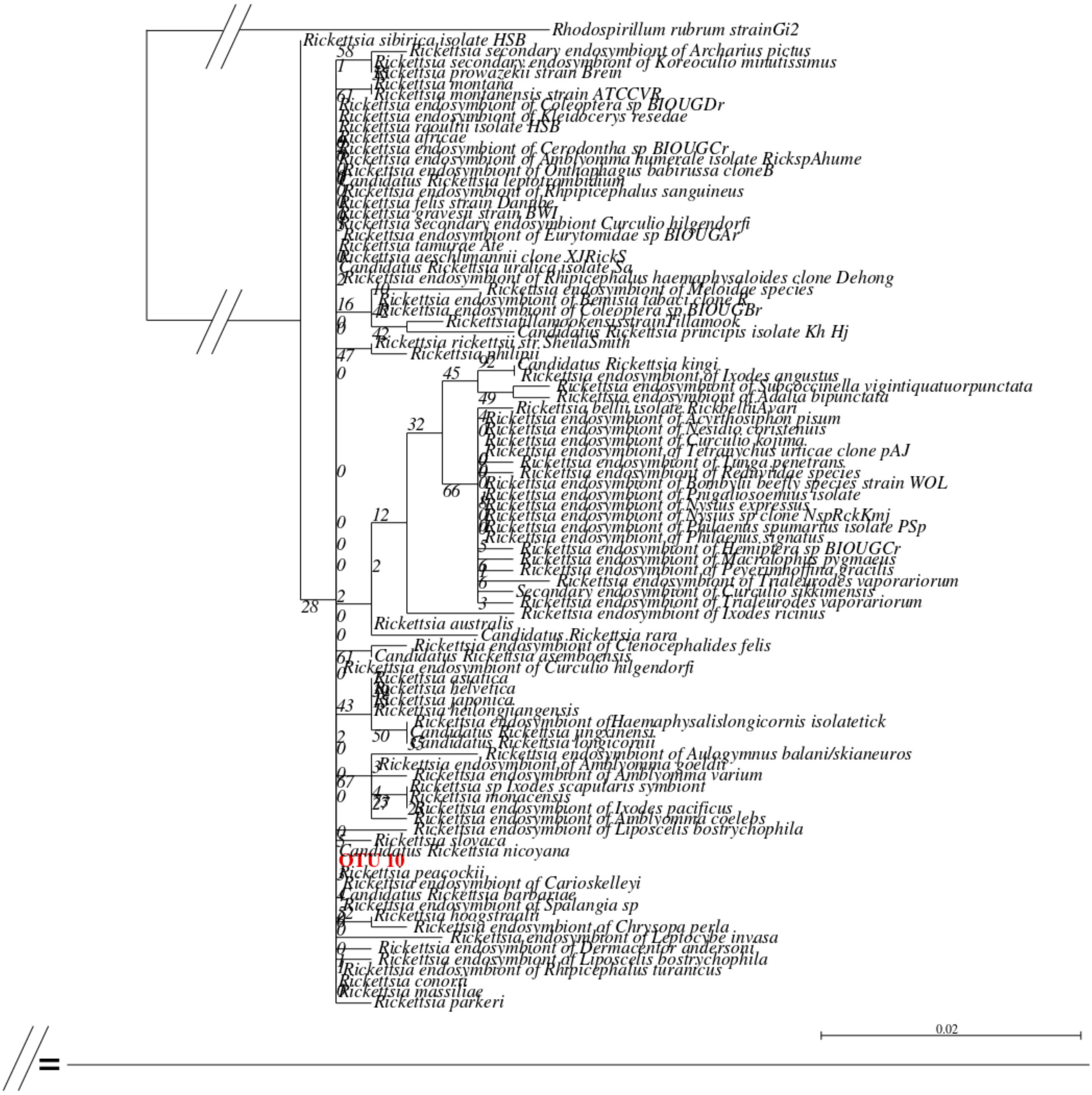

Supplementary Figure S5: Phylogenetic inference on 16S rRNA gene sequences of *Rickettsia* sp. detected in our samples. The sequences obtained for the 16S rRNA gene do not contain sufficient information to allow species assignment of OTU_10 (in red).

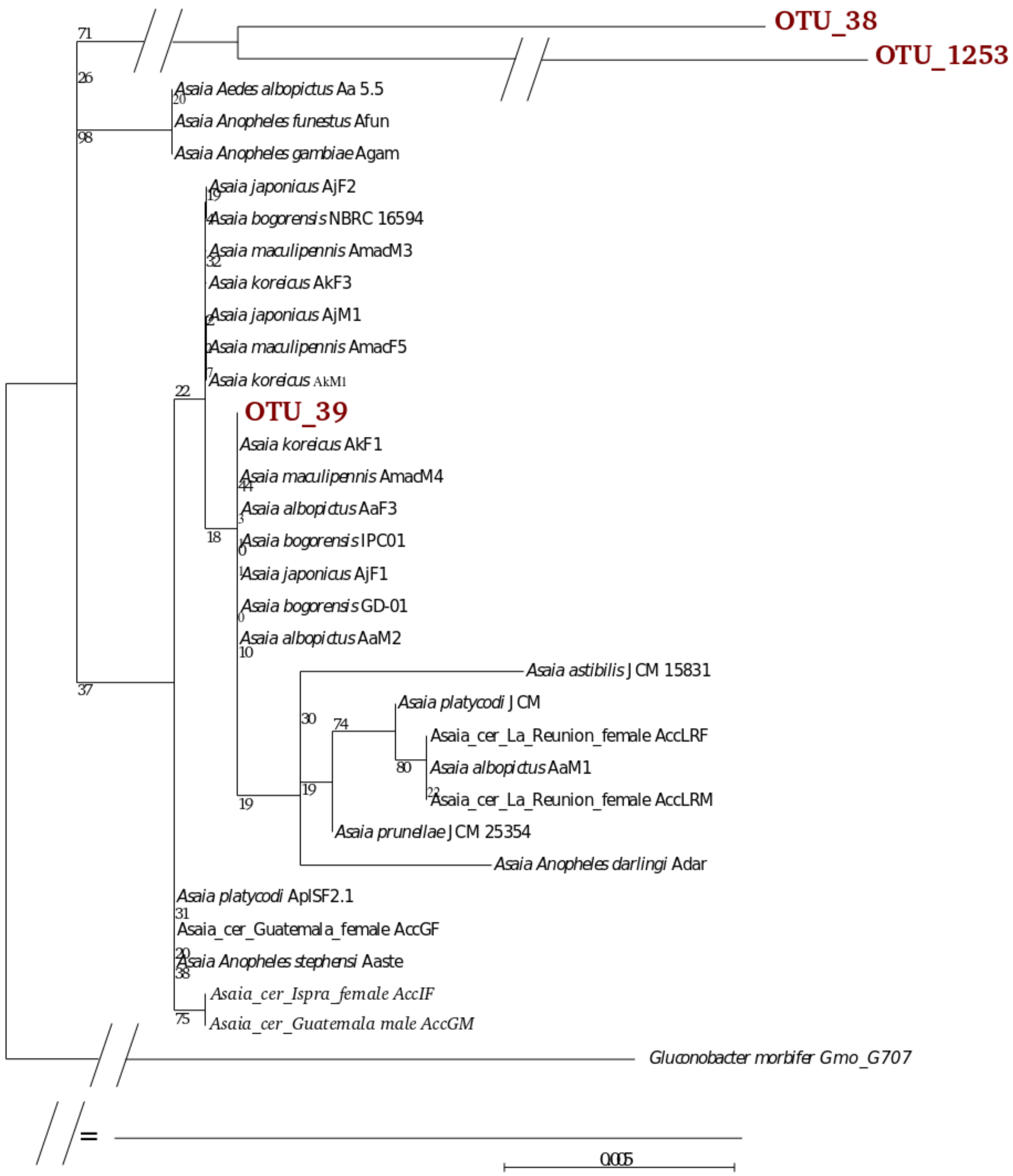

Supplementary Figure S6: Phylogenetic inference of *Asaia* OTUs based on 16s rRNA sequences. Amplicon sequences from our dataset are in red. OTU_39 clusters with *Asaia* strains detected in mosquitoes, whereas OTU_38 and OTU_1253 form long branches and their taxonomy could not be resolved.

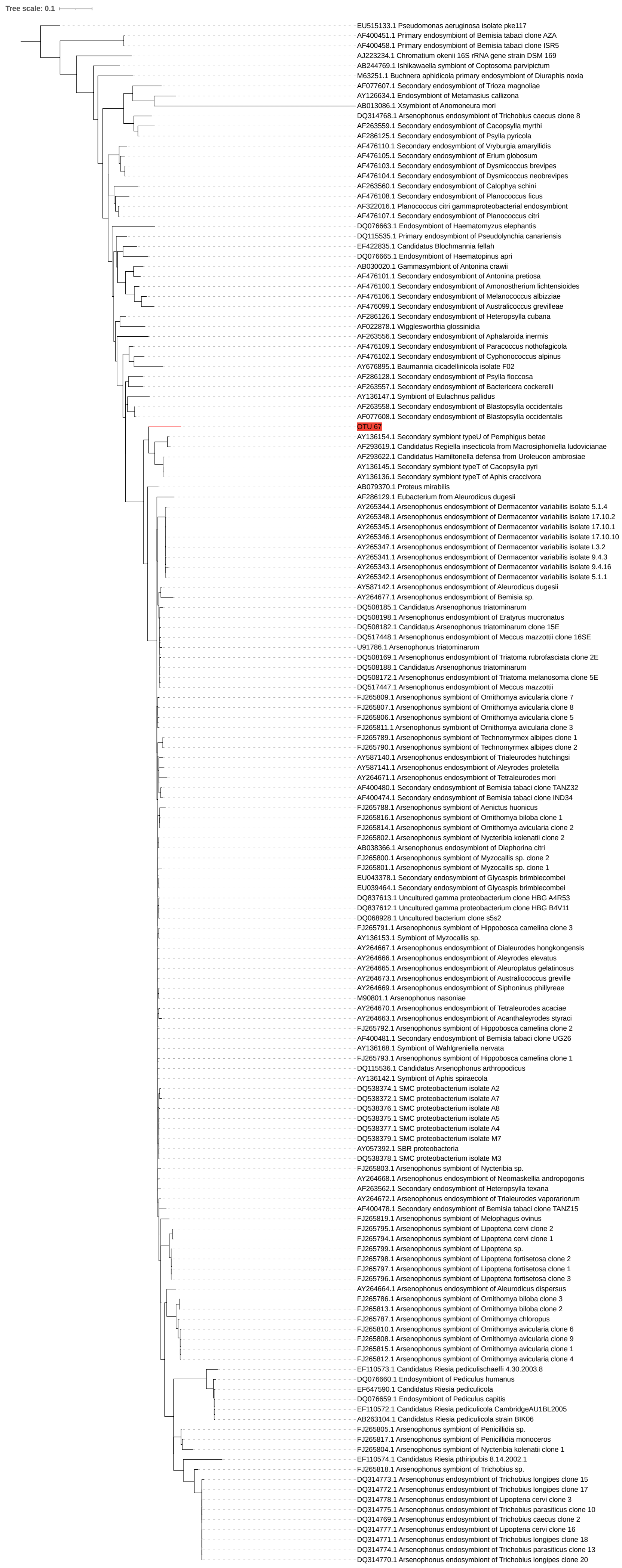
Tree scale: 0.1
EU515133.1 Pseudomonas aeruginosa isolate pke117
AF400451.1 Primary endosymbiont of Bemisia tabaci clone AZA
AF400458.1 Primary endosymbiont of Bemisia tabaci clone ISR5
AJ223234.1 Chromatium okenii 16S rRNA gene strain DSM 169
AB244769.1 Ishikawaella symbiont of Coptosoma parvipictum
M63251.1 Buchnera aphidicola primary endosymbiont of Diuraphis noxia
AF077607.1 Secondary endosymbiont of Trioza magnoliae
AY126634.1 Endosymbiont of Metamasius callizona
AB013086.1 Xsymbiont of Anomoneura mori
DQ314768.1 Arsenophonus endosymbiont of Trichobius caecus clone 8
AF263559.1 Secondary endosymbiont of Cacopsylla myrthi
AF286125.1 Secondary endosymbiont of Psylla pyricola
AF476110.1 Secondary endosymbiont of Vryburgia amaryllidis
AF476105.1 Secondary endosymbiont of Erium globosum
AF476103.1 Secondary endosymbiont of Dysmicoccus brevipes
AF476104.1 Secondary endosymbiont of Dysmicoccus neobrevipes
AF263560.1 Secondary endosymbiont of Calophya schini
AF476108.1 Secondary endosymbiont of Planococcus ficus
AF322016.1 Planococcus citri gammaproteobacterial endosymbiont
AF476107.1 Secondary endosymbiont of Planococcus citri
DQ076663.1 Endosymbiont of Haematomyzus elephantis
DQ115535.1 Primary endosymbiont of Pseudolynchia canariensis
EF422835.1 Candidatus Blochmannia fellah
DQ076665.1 Endosymbiont of Haematopinus apri
AB030020.1 Gammasymbiont of Antonina crawii
AF476101.1 Secondary endosymbiont of Antonina pretiosa
AF476100.1 Secondary endosymbiont of Amonostherium lichtensioides
AF476106.1 Secondary endosymbiont of Melanococcus albizziae
AF476099.1 Secondary endosymbiont of Australicoccus grevilleae
AF286126.1 Secondary endosymbiont of Heteropsylla cubana
AF022878.1 Wigglesworthia glossinidia
AF263556.1 Secondary endosymbiont of Aphalaroida inermis
AF476109.1 Secondary endosymbiont of Paracoccus nothofagicola
AF476102.1 Secondary endosymbiont of Cyphonococcus alpinus
AY676895.1 Baumannia cicadellinicola isolate F02
AF286128.1 Secondary endosymbiont of Psylla floccosa
AF263557.1 Secondary endosymbiont of Bactericera cockerelli
AY136147.1 Symbiont of Eulachnus pallidus
AF263558.1 Secondary endosymbiont of Blastopsylla occidentalis
AF077608.1 Secondary endosymbiont of Blastopsylla occidentalis
OTU 67
AY136154.1 Secondary symbiont typeU of Pemphigus betae
AF293619.1 Candidatus Regiella insecticola from Macrosiphoniella ludovicianae
AF293622.1 Candidatus Hamiltonella defensa from Uroleucon ambrosiae
AY136145.1 Secondary symbiont typeT of Cacopsylla pyri
AY136136.1 Secondary symbiont typeT of Aphis craccivora
AB079370.1 Proteus mirabilis
AF286129.1 Eubacterium from Aleurodicus dugesii
AY265344.1 Arsenophonus endosymbiont of Dermacentor variabilis isolate 5.1.4
AY265348.1 Arsenophonus endosymbiont of Dermacentor variabilis isolate 17.10.2
AY265345.1 Arsenophonus endosymbiont of Dermacentor variabilis isolate 17.10.1
AY265346.1 Arsenophonus endosymbiont of Dermacentor variabilis isolate 17.10.10
AY265347.1 Arsenophonus endosymbiont of Dermacentor variabilis isolate L3.2
AY265341.1 Arsenophonus endosymbiont of Dermacentor variabilis isolate 9.4.3
AY265343.1 Arsenophonus endosymbiont of Dermacentor variabilis isolate 9.4.16
AY265342.1 Arsenophonus endosymbiont of Dermacentor variabilis isolate 5.1.1
AY587142.1 Arsenophonus endosymbiont of Aleurodicus dugesii
AY264677.1 Arsenophonus endosymbiont of Bemisia sp.
DQ508185.1 Candidatus Arsenophonus triatominarum
DQ508198.1 Arsenophonus endosymbiont of Eratyrus mucronatus
DQ508182.1 Candidatus Arsenophonus triatominarum clone 15E
DQ517448.1 Arsenophonus endosymbiont of Meccus mazzottii clone 16SE
U91786.1 Arsenophonus triatominarum
DQ508169.1 Arsenophonus endosymbiont of Triatoma rubrofasciata clone 2E
DQ508188.1 Candidatus Arsenophonus triatominarum
DQ508172.1 Arsenophonus endosymbiont of Triatoma melanosoma clone 5E
DQ517447.1 Arsenophonus endosymbiont of Meccus mazzottii
FJ265809.1 Arsenophonus symbiont of Ornithomya avicularia clone 7
FJ265807.1 Arsenophonus symbiont of Ornithomya avicularia clone 8
FJ265806.1 Arsenophonus symbiont of Ornithomya avicularia clone 5
FJ265811.1 Arsenophonus symbiont of Ornithomya avicularia clone 3
FJ265789.1 Arsenophonus symbiont of Technomyrmex albipes clone 1
FJ265790.1 Arsenophonus symbiont of Technomyrmex albipes clone 2
AY587140.1 Arsenophonus endosymbiont of Trialeurodes hutchingsi
AY587141.1 Arsenophonus endosymbiont of Aleyrodes proletella
AY264671.1 Arsenophonus endosymbiont of Tetraleurodes mori
AF400480.1 Secondary endosymbiont of Bemisia tabaci clone TANZ32
AF400474.1 Secondary endosymbiont of Bemisia tabaci clone IND34
FJ265788.1 Arsenophonus symbiont of Aenictus huonicus
FJ265816.1 Arsenophonus symbiont of Ornithomya biloba clone 1
FJ265814.1 Arsenophonus symbiont of Ornithomya avicularia clone 2
FJ265802.1 Arsenophonus symbiont of Nycteribia kolenatii clone 2
AB038366.1 Arsenophonus endosymbiont of Diaphorina citri
FJ265800.1 Arsenophonus symbiont of Myzocallis sp. clone 2
FJ265801.1 Arsenophonus symbiont of Myzocallis sp. clone 1
EU043378.1 Secondary endosymbiont of Glycaspis brimblecombei
EU039464.1 Secondary endosymbiont of Glycaspis brimblecombei
DQ837613.1 Uncultured gamma proteobacterium clone HBG A4R53
DQ837612.1 Uncultured gamma proteobacterium clone HBG B4V11
DQ068928.1 Uncultured bacterium clone s5s2
FJ265791.1 Arsenophonus symbiont of Hippobosca camelina clone 3
AY136153.1 Symbiont of Myzocallis sp.
AY264667.1 Arsenophonus endosymbiont of Dialeurodes hongkongensis
AY264666.1 Arsenophonus endosymbiont of Aleyrodes elevatus
AY264665.1 Arsenophonus endosymbiont of Aleuroplatus gelatinosus
AY264673.1 Arsenophonus endosymbiont of Australiococcus greville
AY264669.1 Arsenophonus endosymbiont of Siphoninus phillyreae
M90801.1 Arsenophonus nasoniae
AY264670.1 Arsenophonus endosymbiont of Tetraleurodes acaciae
AY264663.1 Arsenophonus endosymbiont of Acanthaleyrodes styraci
FJ265792.1 Arsenophonus symbiont of Hippobosca camelina clone 2
AF400481.1 Secondary endosymbiont of Bemisia tabaci clone UG26
AY136168.1 Symbiont of Wahlgreniella nervata
FJ265793.1 Arsenophonus symbiont of Hippobosca camelina clone 1
DQ115536.1 Candidatus Arsenophonus arthropodicus
AY136142.1 Symbiont of Aphis spiraecola
DQ538374.1 SMC proteobacterium isolate A2
DQ538372.1 SMC proteobacterium isolate A7
DQ538376.1 SMC proteobacterium isolate A8
DQ538375.1 SMC proteobacterium isolate A5
DQ538377.1 SMC proteobacterium isolate A4
DQ538379.1 SMC proteobacterium isolate M7
AY057392.1 SBR proteobacteria
DQ538378.1 SMC proteobacterium isolate M3
FJ265803.1 Arsenophonus symbiont of Nycteribia sp.
AY264668.1 Arsenophonus endosymbiont of Neomaskellia andropogonis
AF263562.1 Secondary endosymbiont of Heteropsylla texana
AY264672.1 Arsenophonus endosymbiont of Trialeurodes vaporariorum
AF400478.1 Secondary endosymbiont of Bemisia tabaci clone TANZ15
FJ265819.1 Arsenophonus symbiont of Melophagus ovinus
FJ265795.1 Arsenophonus symbiont of Lipoptena cervi clone 2
FJ265794.1 Arsenophonus symbiont of Lipoptena cervi clone 1
FJ265799.1 Arsenophonus symbiont of Lipoptena sp.
FJ265798.1 Arsenophonus symbiont of Lipoptena fortisetosa clone 2
FJ265797.1 Arsenophonus symbiont of Lipoptena fortisetosa clone 1
FJ265796.1 Arsenophonus symbiont of Lipoptena fortisetosa clone 3
AY264664.1 Arsenophonus endosymbiont of Aleurodicus dispersus
FJ265786.1 Arsenophonus symbiont of Ornithomya biloba clone 3
FJ265813.1 Arsenophonus symbiont of Ornithomya biloba clone 2
FJ265787.1 Arsenophonus symbiont of Ornithomya chloropus
FJ265810.1 Arsenophonus symbiont of Ornithomya avicularia clone 6
FJ265808.1 Arsenophonus symbiont of Ornithomya avicularia clone 9
FJ265815.1 Arsenophonus symbiont of Ornithomya avicularia clone 1
FJ265812.1 Arsenophonus symbiont of Ornithomya avicularia clone 4
EF110573.1 Candidatus Riesia pediculischaeffi 4.30.2003.8
DQ076660.1 Endosymbiont of Pediculus humanus
EF647590.1 Candidatus Riesia pediculicola
DQ076659.1 Endosymbiont of Pediculus capitis
EF110572.1 Candidatus Riesia pediculicola CambridgeAU1BL2005
AB263104.1 Candidatus Riesia pediculicola strain BIK06
FJ265805.1 Arsenophonus symbiont of Penicillidia sp.
FJ265817.1 Arsenophonus symbiont of Penicillidia monoceros
FJ265804.1 Arsenophonus symbiont of Nycteribia kolenatii clone 1
EF110574.1 Candidatus Riesia pthiripubis 8.14.2002.1
FJ265818.1 Arsenophonus symbiont of Trichobius sp.
DQ314773.1 Arsenophonus endosymbiont of Trichobius longipes clone 15
DQ314772.1 Arsenophonus endosymbiont of Trichobius longipes clone 17
DQ314778.1 Arsenophonus endosymbiont of Lipoptena cervi clone 3
DQ314775.1 Arsenophonus endosymbiont of Trichobius parasiticus clone 10
DQ314769.1 Arsenophonus endosymbiont of Trichobius caecus clone 2
DQ314777.1 Arsenophonus endosymbiont of Lipoptena cervi clone 16
DQ314771.1 Arsenophonus endosymbiont of Trichobius longipes clone 18
DQ314774.1 Arsenophonus endosymbiont of Trichobius parasiticus clone 13
DQ314770.1 Arsenophonus endosymbiont of Trichobius longipes clone 20

Supplementary Figure S7: Phylogenetic inference on 16S rRNA gene sequences of *Morganellaceae* detected in our samples. The sequences obtained for the 16S rRNA gene do not contain sufficient information to allow species assignment, although they place OTU_67 (in red) as sister group of symbionts of aphids.
